# Predictive diagnostic value for the clinical features accompanying intellectual disability in children with pathogenic copy number variations: a multivariate analysis

**DOI:** 10.1186/1824-7288-40-39

**Published:** 2014-04-28

**Authors:** Elisa Caramaschi, Ilaria Stanghellini, Pamela Magini, Maria Grazia Giuffrida, Silvia Scullin, Tiziana Giuva, Patrizia Bergonzini, Azzurra Guerra, Paolo Paolucci, Antonio Percesepe

**Affiliations:** 1 Pediatrics Unit, Department of Mother & Child, University Hospital of Modena, Modena, Italy; 2 Medical Genetics Unit, Department of Mother & Child, University Hospital of Modena, Via del Pozzo, 71, 41124 Modena, Italy; 3 Medical Genetics Unit, Department of Medical and Surgical Sciences, University of Bologna, Bologna, Italy; 4 Mendel Laboratory, IRCCS “Casa Sollievo della Sofferenza” Hospital, S. Giovanni Rotondo, Italy; 5 Pediatrics Unit, B. Ramazzini Hospital, Carpi, Italy

**Keywords:** Array CGH, CNVs, Developmental delay/intellectual disability, Malformations, Multivariate analysis

## Abstract

**Background:**

Array comparative genomic hybridization (a-CGH) has become the first-tier investigation in patients with unexplained developmental delay/intellectual disability (DD/ID). Although the costs are progressively decreasing, a-CGH is still an expensive and labour-intensive technique: for this reason a definition of the categories of patients that can benefit the most of the analysis is needed. Aim of the study was to retrospectively analyze the clinical features of children with DD/ID attending the outpatient clinic of the Mother & Child Department of the University Hospital of Modena subjected to a-CGH, to verify by uni- and multivariate analysis the independent predictors of pathogenic CNVs.

**Methods:**

116 patients were included in the study. Data relative to the CNVs and to the patients’ clinical features were analyzed for genotype/phenotype correlations.

**Results and conclusions:**

27 patients (23.3%) presented pathogenic CNVs (21 deletions, 3 duplications and 3 cases with both duplications and deletions). Univariate analysis showed a significant association of the pathogenic CNVs with the early onset of symptoms (before 1 yr of age) and the presence of malformations and dysmorphisms. Logistic regression analysis showed a significant independent predictive value for diagnosing a pathogenic CNV for malformations (P = 0.002) and dysmorphisms (P = 0.023), suggesting that those features should address a-CGH analysis as a high-priority test for diagnosis.

## Background

The use of array-CGH (a-CGH) has recently become a mainstay for the diagnosis of a broad spectrum of disorders, including developmental delay/intellectual disability (DD/ID), malformations and dysmorphisms, due to its higher resolution power (about 100-fold) and diagnostic yield (5- to 10-fold) compared with the classical karyotype [1-3]. Large scale genomic analysis have highlighted a pathogenic role for many copy number variations (CNVs), which have been detected both in cases with syndromic features and in others lacking a clinical hallmark pointing to a specific genetic condition [4,5]. In addition, about 12% of a healthy individual's genome can contain a copy number variation whose role, if any, remains unknown [6]. Dedicated on-line databases (DatabasE of Chromosomal Imbalance and Phenotype in Humans using Ensemble Resources – DECIPHER- http://decipher.sanger.ac.uk; Database of Genomic Variants –DGV- http://projects.tcag.ca/variation/), continuously expanded by the scientific community, are providing growing information to perform the genotype/phenotype correlations.

Although the costs are progressively decreasing, a-CGH is still an expensive and labour- intensive technique, and as such, cost-, clinical impact- and genotype/phenotype-analysis have tried to define the convenience and the correct indications to perform the analysis in selected categories of patients [7]. For example, the presence of pathogenic CNVs has been correlated with a severe clinical presentation or with a pleiotropic expression of the disease [5,8,9]; other studies have demonstrated that the presence of at least two clinical features increases the likelihood that the phenotype is associated with CNVs [10], although many exceptions exist, which underlie the extreme phenotypic heterogeneity of genomic disorders.

To highlight the most useful indications to perform the a-CGH analysis in children with DD/ID and associated clinical features (i.e. malformations, epilepsy, dysmorphisms), we report a retrospective study based on 116 consecutive cases referred to the Department of Mother & Child of the University Hospital of Modena. The distribution of several clinical features was studied by univariate and multivariate analysis in patients with vs. those without pathogenic CNVs, to identify the strongest predictors of the presence of genomic rearrangements and to recognize those cases in which a-CGH would be crucial for achieving the diagnosis.

## Methods

### The clinic

Patients have been recruited at the outpatient Pediatric Clinic of the Mother & Child Department of the Modena University Hospital, in the years 2006–2013. The clinic receives patients across the city area (about 600,000 inhabitants) and offers second-level assistance to the Community Support Services. The outpatients are sequentially evaluated by paediatricians, paediatric neurologists and medical geneticists and their clinical information, including previous personal and familial medical history, are collected. Verification of the reported diagnosis is done through the consultation of the medical records. Patients’ follow-up consists of annual clinical evaluations during which the diagnostic process is carried out by using traditional clinical and instrumental tools and genetic testing, when appropriate.

### The patients

For the purposes of the study we selected consecutive paediatric patients undergoing a-CGH analysis for the presence of DD/ID associated with at least one of the following clinical features: 1) malformations, 2) epilepsy, 3) dysmorphisms. Malformations were defined as major defects (i.e. those affecting organs like the heart, the urogenital tract), whereas isolated minor congenital anomalies (i.e. persistent foramen ovale) were not considered for the purposes of the study.

Clinical and genetic data obtained were retrospectively collected in an Excel format database, including patient’s records, pregnancy, neonatal and family histories, body measurements, neurologic examination, brain imaging, specialist opinions (eg. radiologist, surgeon), conventional karyotype with a resolution of 550 bands for aploid set [11] and a-CGH results (number, type, size, inheritance of CNVs).

### Array-CGH analysis

The Agilent 44 K platform was used for the analysis of all patients, following manufacturer’s instructions (Agilent Technologies). Briefly, 500 ng of patient and control DNAs were double-digested with restriction enzymes (AluI and RsaI) and differentially labeled with Cy-5 and Cy-3, respectively. A “loop” strategy with three phenotypically different patients was used (for example A vs. B, B vs. C and C vs. A). After hybridization on the 44 K array, slides were washed and scanned. Agilent Feature Extraction and Genomic Workbench softwares were used to calculate log ratios, to create a graphical visualization of the results and to call copy number aberrations (ADM-1 algorithm - threshold 6.0 -). Changes of 3 or more consecutive oligonucleotides with the same log ratio (deletions about −1 or duplications about +0.5) were called as CNVs. The loop strategy allowed the simultaneous confirmation of each CNV, which had to be present in two arrays of the loop with opposite values of log ratio and the elimination of most of the polymorphic CNVs with high frequency in the population.

CNVs were compared to the DECIPHER, DGV, ISCA (International Standard for Cytogenomic Arrays consortium, https://www.iscaconsortium.org/index.php/search) and Troina Database of Human CNVs (http://gvarianti.homelinux.net/gvariantib37/index.php) and classified pathogenic, likely pathogenic, benign, likely benign or of unknown significance, using the following criteria:

•pathogenic: anomalies mapping on genomic regions associated to known syndromes or involving known dosage-sensitive genes and large imbalances of *de novo* origin or inherited from a similarly affected parent;

•likely pathogenic: small alterations of *de novo* origin or inherited from a parent with a similar phenotype, involving genomic regions or genes whose possible association with clinical conditions has not been definitely identified, but could be supposed from the clinical databases (DECIPHER, ISCA and Troina);

•benign: polymorphic variants reported in several healthy individuals in more than one study within DGV and/or alterations detected in at least two patients with clearly distinct phenotypes of the present cohort;

•likely benign: microdeletions and microduplications reported in few controls in DGV, but defined benign or likely benign in the clinical databases (DECIPHER, ISCA and Troina) and inherited from a normal parent;

•of unknown significance: inherited alterations not described or with discordant definitions among those databases [12].

### Statistical analysis

Patients were stratified according to the presence of several clinical features, all potentially related to the presence of a pathological phenotype (positive family history, delivery before 37 gestational weeks, apgar score <7, low birth weight – less than the fifth centile-, early onset of symptoms (<1 year of age), motor delay, dysmorphisms, malformations –brain excluded-, speech delay, epilepsy, cerebral malformations), converting descriptive variables into numerical. For the purposes of the analysis pathogenic and likely pathogenic CNVs were grouped together. The relationship between each variable and the presence of pathogenic CNVs was first analyzed by means of univariate analysis (chi-squared for 2-by-2 tables). Furthermore, in order to identify the independent predictors of the diagnosis of pathogenic CNVs, the variables resulting significant at the univariate analysis were subjected to logistic regression, using the presence of pathogenic CNVs as a dependent dichotomous variable [13], and odds ratios and their 95% confidence intervals (CI) were calculated. Results were considered statistically significant for p < 0.05. Statistical analyses were carried out with STATA software (version 11.1, 2010; StataCorp, College Station, TX, USA).

The study was approved by Modena Institutional Review Board (Protocol No. 249/12, 5^th^ March 2013).

## Results

Out of the 116 patients (58 males and 58 females) subjected to a-CGH analysis, 111 had a normal and 5 an abnormal karyotype (in those latter cases a-CGH was used to characterize the genomic alteration). Abnormal karyotypic findings are listed in Table 1.

**Table 1:** Abnormal karyotypes found in patients who underwent a-CGH for the characterization of the abnormality

**Patient No.**	**Karyotype**
5	47,XX,del(6)(q24),+mar[30]/46,XX,del(6)(q24)[15]
12	45,XY,t(5;15)(p15.3;q11.2)
25	46,XY,dup(19)(q12q13.2)
6	46,XY,der(9)t(1;9)(q41;q34)
26	46,XY,r(22).ish del(22q13)

For their a-CGH results refer to Table 3.

Table 2 shows the number, type, inheritance and clinical interpretation of the CNVs found in the study population: 41 patients showed at least one CNV, whereas 75 did not show any. Twenty-seven out of 41 patients carried a pathogenic (or likely pathogenic) CNV, whereas 12 had a benign one. In 2 unrelated cases (both had a 489 Kb duplication in 15q13.3 of maternal inheritance) the role of the CNV found is unknown. The genomic details of the pathogenic CNVs and their clinical correlates are listed in Table 3.

**Table 2:** Type, number, size and inheritance of the CNVs found according to the clinical interpretation

**Variable**	**Patients with pathogenic CNVs**	**Patients with benign CNVs**	**Patients with unknown CNVs**
	**N = 27**	**N = 12**	**N = 2**
**Type**			
Deletion	21 (77.8%)	2 (16.7%)	
Duplication	3 (11.1%)	9 (75.0%)	2 (100%)
Duplication and deletion	3 (11.1%)	1 (8.3%)	
**No. CNVs**			
1	22	8	2
≥2	5	4	
**Size**			
≤0.5 Mb		6 (50%)	2 (100%)
>0.5 Mb	27 (100%)	6 (50%)	
**Inheritance**			
De novo	21 (77.8%)	1 (8.3%)	
Familial	5 (18.5%)	7 (58.3%)	2 (100%)
Unknown	1 (3.7%)	4 (33.3)	

**Table 3:** List of genomic rearrangements in patients with pathogenic CNVs

**Patient No.**	**Sex**	**Type**	**Locus**	**Genomic coordinates**	**Inheritance**	**Syndrome**	**OMIM**
1	M	del	1p36.33p36.31	chr1:837,491-6,458,739	de novo	1p36.33 deletion S.	607872
2	F	del	1p36.33p36.31	chr1:544,268-5,983,997	de novo	1p36.33 deletion S.	607872
3	F	del	1p36.22p36.13	chr1:11,722,823-17,104,536	de novo	-	
		del	1p35.1p34.1	chr1:31,080,722-32,355,071	de novo	-	
4	F	del	1q23.3q24.2	chr:162,345,741-169,317,061	de novo	-	
5	F	del	1q41	chr1:217,316,641-218,402,514	not known	1q41-1q42 deletionS.	612530
		del	6q24.2	chr6:144,223,274-144,446,997	not known	-	
6 Ref [14]	M	dup	1q42qter	chr1:223,858,274-248,105,710	de novo	-	
7	F	del	2q14.3	chr2:125,049,268-129,322,082	de novo	-	
8	F	del	2q24.3	chr2:166,198,780-166,930,047	de novo	-	
9	M	dup	4p15.32	chr4:26,509-27,414	de novo	-	
		del	17q24.2	chr17:64,682,538-65,991,538	de novo	-	
10	M	del	4q26q28.1	chr4:117,685,833-127,471,713	de novo	-	
11	M	dup	4q33q35.1	chr4:171,936,073-186,883,667	de novo	-	
		del	4q35.1q35.2	chr4:186,913,016-190,976,417	de novo	-	
12 Ref [15]	M	del	5p15.33p15.32	chr5:22,178-5,539,182	paternal	Cri du Chat S.	123450
13	F	del	5q14.3q15	chr5:89,535,781-92,554,566	de novo	-	
14	M	del	5q23.2q31.1	chr5:124,391,181-134,632,894	de novo	-	
15	F	del	6q27	chr6:168,378,740-169,862,121	de novo	-	
16	M	del	7q11.23	chr7:72,039,051-73,771,238	de novo	Williams-Beuren S.	194050
17	M	del	7q21.13q21.3	chi7:89,993,838-96,278,971	de novo	Split Hand Foot Malformation 1 with sensorineural hearing loss	220600
18	M	del	7q31.1q31.2	chr7:113,824,764-115,669,764	de novo	-	
19	F	del	7q36	chr7:153,669,067-159,107,239	de novo	Holoprosencephaly-3	142945
20	F	del	15q13.2q13.3	chr15:31,014,000-32,510,000	maternal	-	
21	F	del	15q26.3	chr15:99,836,103-102,351,195	paternal	15q26-qter deletion S.	612626
22	F	dup	16p13.11p12.3	chr16:15,492,317-17,804,366	maternal	-	
23	F	del	17q12	chr17:31,891,355-33,726,698	de novo	17q12 deletion S	614527
24	M	del	19p13.3	chr19:64,447-721,353	de novo	-	
25 Ref [16]	M	dup	19q12q13.2	chr19:28,272,160-40,699,160	de novo	-	
26	M	del	22q13.31	chr22:45,576,757-51,178,264	de novo	Phelan Mc Dermid S.	606232
		dup	Xq28	chrX:153,322,653-153,406,233	de novo	-	
27	F	del	22q11.21	chr22:19,375,985-21,382,953	maternal	DiGeorge S.	188400

Genomic coordinates refer to Human Genome Assembly (Hg 19). If the analysis was performed with previous assemblies, data were converted in Hg 19. M = male, F = female, del = deletion, dup = duplication.

**Figure 1:**
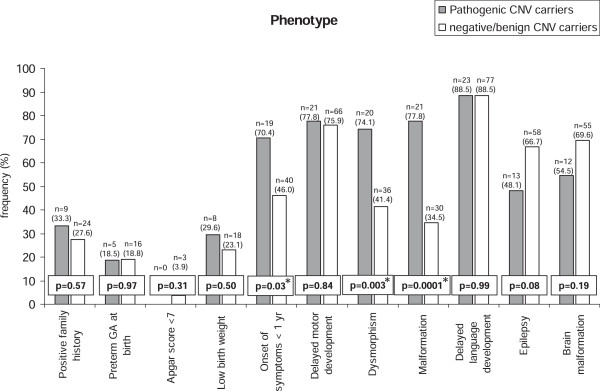
**Clinical features of the patients under study according to the presence of pathogenic CNVs.** For each variable the number (n=), the percentage of patients (in parenthesis) and the P values (in boxes) are reported. Statistical significance is indicated with the presence of an asterisk.

By comparing the frequency of each clinical feature in patients with pathogenic vs. those with benign or absent CNVs (the 2 cases with CNVs of unknown interpretation were excluded), a statistically significant association with the presence of pathogenic CNVs was found for the early onset of symptoms (p = 0.027), the presence of dysmorphisms (p = 0.003) and malformations (p = 0.0001), whereas all the other variables did not show any statistical significance (Figure 1).

The clinical variables found to be associated with pathogenic CNVs were further analyzed by a multivariate analysis and a statistically significant odds ratio for association with pathogenic CNVs was confirmed for the presence of dysmorphisms (p = 0.023) and malformations (p = 0.002), whereas the onset of symptoms before the first year of age was not (p = 0.069) (Table 4).

**Table 4:** Multivariate analysis for the clinical features significantly associated to the pathogenic CNVs at the univariate analysis

**Clinical variables**	**Odds ratio**	**Std. Err.**	**P**	**[95% conf. interval]**
Early onset of symptoms (before 1 yr of age)	2.567	1.331	0.069	0.930 - 7.090
Dysmorphisms	3.326	1.752	0.023	1.184 - 9.340
Malformations	5.365	2.871	0.002	1.880 - 15.312

## Discussion

The use of a-CGH in the clinical settings has been shown to improve the follow-up, the rehabilitation strategies and, in selected cases, the prophylactic therapy in patients in which the presence of pathogenic CNVs is demonstrated [17,18].

Although the utility is already proven, the Health Care System of the Emilia-Romagna Region has introduced a-CGH in the list of reimbursable tests only in 2013 and the network of laboratories in the Region is still organizing to offer the analysis with short waiting lists and lead times. For all these reasons, understanding which patients and families may benefit the most is required and clinical predictors are needed. Although evidence already exists to offer a-CGH as a first-tier exam in patients showing ID with or without additional clinical features [1], further data have shown that patients with syndromic ID (multiple congenital anomalies) or with severe phenotypes have a higher likelihood to be carriers of CNVs [10].

In our study, the relative burden conveyed by each feature accompanying DD/ID was evaluated by a multivariate analysis on 116 patients in which the frequency of several clinical variables referring to the pregnancy, delivery, family history, associated malformations, psychomotor development and dysmorphisms were compared between patients showing pathogenic CNVs vs. all the others (those negative and with benign CNVs).

CNVs were detected in 41 out of 116 patients (35.3%) and a pathogenic role was attributed in 27 cases (23.3% of the study population). Among those latter, 21 had a de novo rearrangement, 5 were inherited (Table 3) (the transmitting parent always displayed some degree of phenotype) and 1 was of unknown origin. On the other hand, among the benign CNVs carriers only 1 patient presented a de novo rearrangement, a 500 Kb interstitial duplication in 14q11.2 of unequivocal interpretation, due to the high frequency (>3%) in the normal population [19]. Deletions were 85% of the pathogenic CNVs, consistent with the notion that haploinsufficiencies are less tolerated than duplications in the human genome [20].

When analysing the clinical features associated with DD/ID and their relative frequency in patients with or without pathogenic CNVs, no difference emerged for birth-related variables, delay in motor or language development or for brain malformations and epilepsy (Figure 1), which are possibly attributable also to different aetiologies (i.e. monogenic or multifactorial), due to their high genetic heterogeneity. On the other hand, the onset of symptoms before one year of life (p = 0.027), the presence of malformations (p = 0.0001) and of dysmorphisms (p = 0.003) resulted significantly associated to the pathogenic CNVs at the univariate analysis (Figure 1), confirming previous data referred to other European populations [10]. When subjected to logistic regression analysis, the dysmorphisms (p = 0.023) and the malformations (p = 0.002) emerged as independent predictors of diagnosing a pathogenic CNV in children with DD/ID, whereas the early onset of symptoms, an additional indicator of the gravity of the phenotype encompassing neonatal hypotonia, infantile epilepsy and motor delay, failed to show a significant result, possibly due to a type 2 statistical error caused by the low number of observations.

Our results confirm that severe phenotypes characterized by the presence of malformations and dysmorphisms associated with DD/ID are causally related to the presence of CNVs, as previously demonstrated [10,21]; moreover, the analysis predicts the likelihood to detect a pathogenic genomic alteration, attributing a 3.3-fold increase to the presence of dysmorphic features and a 5.3-fold to the malformations (Table 4), thus reaffirming the importance of a thorough phenotypic characterization of the patients undergoing a-CGH analysis for maximizing the results [10].

## Conclusions

In conclusion, dissecting the phenotype of children with DD/ID undergoing a-CGH led us to identify the malformations and the dysmorphisms as independent clinical predictors for finding pathogenic CNVs, indicating that the presence of those features in association with DD/ID should address a-CGH analysis as a high-priority test for diagnosis.

## Competing interests

The authors declare that they have no competing interests.

## Authors’ contributions

EC collected the data, organized the database, participated in data analysis; IS drafted the manuscript, participated in data analysis and critical discussion; PM, MGG, SS, TG, PB participated in data collection; AG, PP participated in critical discussion, AP conceived the study, participated in data analysis, manuscript drafting and critical discussion. All authors approved the final version of the manuscript.
